# The Use of ^18^F-FDG PET/CT Metabolic Parameters in Predicting Overall Survival in Patients Undergoing Restaging for Malignant Melanoma

**DOI:** 10.3390/diagnostics12030595

**Published:** 2022-02-25

**Authors:** Khanyisile N. Hlongwa, Kgomotso M. G. Mokoala, Zvifadzo Matsena-Zingoni, Mariza Vorster, Mike M. Sathekge

**Affiliations:** 1 Department of Nuclear Medicine, University of Pretoria and Steve Biko Academic Hospital, Pretoria 0001, South Africa; khanyi29@gmail.com (K.N.H.); kgomotso.mokoala@up.ac.za (K.M.G.M.); marizavorster@gmail.com (M.V.); 2 Division of Epidemiology and Biostatistics, School of Public Health, University of Witwatersrand, Johannesburg 2193, South Africa; zvifadzo.matsenazingoni@wits.ac.za; 3 Nuclear Medicine Research Infrastructure (NuMeRI), Steve Biko Academic Hospital, Pretoria 0001, South Africa

**Keywords:** ^18^F-FDG PET/CT (fluorodeoxyglucose positron emission tomography/computed tomography), metabolic parameters, metabolic tumor volume (MTV), total lesion glycolysis (TLG), maximum standard uptake value (SUVmax), oncology, malignant melanoma (MM), overall survival (OS), restaging

## Abstract

Malignant melanoma is one of the more aggressive cancers in the skin, with an increasing incidence every year. Melanoma has a better prognosis if diagnosed early and survival tends to decrease once the disease has metastasized. Positron emission tomography (PET) with 2-[^18^F]fluoro-2-deoxy-D-glucose (^18^F-FDG) has been used extensively over the past two decades in staging and assessing responses to therapy in patients with melanoma. Metabolic PET parameters have been demonstrated to be independent prognostic factors for progression-free survival (PFS) and overall survival (OS) in different malignancies, melanoma included. In our study, we evaluated the metabolic parameters of ^18^F-FDG PET/CT (flourodeoxyglucose positron emission tomography/computed tomography) in predicting the overall survival in patients with malignant melanoma who presented for restaging. Metabolic PET parameters (maximum standardized uptake value (SUVmax), metabolic tumor volume (MTV) and total lesion glycolysis (TLG)) of the primary tumor, as well as whole-body MTV and TLG of the metastatic disease, were measured. Survival curves for OS were constructed and mortality rates were determined using the different PET variables. Forty-nine patients who presented for a PET/CT restaging in melanoma were included in this study. We found that non-survivors had significantly higher median MTV (11.86 cm^3^ vs. 5.68 cm^3^; *p*-value = 0.022), TLG (3125 vs. 14; *p*-value = 0.0357), whole-body MTV (53.9 cm^3^ vs. 14.4 cm^3^; *p*-value = 0.0076) and whole-body TLG (963.4 vs. 114.6; *p*-value = 0.0056). This demonstrated that high MTV and TLG values of the primary tumor and whole-body TLG as quantified by ^18^F-FDG PET/CT were prognostic factors for overall survival. The findings may potentially guide clinicians in decision making and identifying patients with a poorer prognosis.

## 1. Introduction

Melanoma is a malignant tumor that arises from the uncontrolled and rapid growth of the melanocytes, which are the pigment-producing cells of the body [1]. The most common form is cutaneous melanoma; however, the tumor can occur in mucosal surfaces, the eye or the brain [1]. Malignant melanoma is one of the aggressive cancers in the skin, with an increasing incidence every year. It is known to represent a small proportion of all cutaneous malignancies but causes a higher rate of fatalities in comparison to other deaths related to skin cancer [1]. Melanoma has a better prognosis if diagnosed early and survival tends to decrease once the disease has metastasized. The cost of management of melanoma contributes significantly to public health and several strategies are being implemented worldwide to improve outcomes via prevention, assessing the at-risk population and improving management strategies [1].

In the South African setting, a country with a diverse population, melanoma is commonly seen in fair-skinned people rather than darker individuals. A retrospective observational study done between 2005 and 2013 from the national cancer registry found that the incidence of melanoma was 2.7 per 100,000 [2]. The different population groups are affected by varying histological subtypes of melanoma, with the superficial spreading subtype being more common in fair individuals and the acral lentiginous subtype seen more in pigmented individuals [2].

Positron emission tomography (PET) with 2-[^18^F]fluoro-2-deoxy-D-glucose (^18^F-FDG) has been used extensively over the past two decades in staging and assessing responses to therapy in patients with melanoma; this has been due to a significant relationship between ^18^F-FDG uptake and glucose metabolism [3]. Flourodeoxyglucose positron emission tomography/computed tomography (^18^F-FDG PET/CT) has been demonstrated to be more reliable in the assessment of survival prognosis in comparison to morphological staging. This is because ^18^F-FDG uptake demonstrates malignant potential, which has been associated with reduced survival [4].

^18^F-FDG uptake has been demonstrated as an excellent substrate in measuring the malignant potential for melanoma; when compared to immunohistochemistry, it demonstrated a positive correlation between glucose transporters (GLUT)-1 and GLUT-3, which is the mechanism that ^18^F-FDG uses to demonstrate malignant tissues in patients with melanoma [5].

The sensitivity, specificity and accuracy of ^18^F-FDG PET to detect metastatic and distant melanoma range from 70 to 100% [6]. The findings in ^18^F-FDG PET studies have been shown to better stage patients, guide further management and provide a better prognosis of patients. This was demonstrated in a study by Reinhardt et al. whereby 250 patients imaged with ^18^F-FDG PET/CT demonstrated more nodal and visceral metastases in comparison to CT alone [6]. A smaller study in South Africa also demonstrated how ^18^F-FDG PET/CT altered staging in patients with malignant melanoma, which further changed the management by treating physicians [7].

Recently, the use of metabolic parameters of ^18^F-FDG PET/CT has been investigated as a tool to risk-stratify patients and to determine prognostic value in patients prior to management, either by surgery or immune therapy, or to detect recurrence [8,9,10,11].

In this study, we investigated the association of metabolic parameters of ^18^F-FDG PET/CT in patients with melanoma undergoing restaging with overall survival.

## 2. Materials and Methods

### 2.1. Patients

^18^F-FDG PET/CT scans of patients presenting for restaging of melanoma were retrospectively reviewed.

Patients were included in the study if they had a suspicion of recurrent or progressive disease as deemed by the referring clinician. The decision to refer a patient for an ^18^F-FDG PET/CT scan and the frequency of imaging were at the discretion of the managing physician. Patients were excluded if they had no disease demonstrable on ^18^F-FDG PET/CT, had advanced primary cancers other than melanoma, were below 18 years of age and had incomplete records.

This retrospective study was approved by the Research Ethics Committee, University of Pretoria (Reference no. 875/2020) and was carried out in accordance with the Declaration of Helsinki.

### 2.2. ^18^F-FDG PET/CT Imaging

Imaging was acquired on a dedicated PET/CT scanner (Biograph 40, Siemens). Standard patient preparation was observed. All patients had a minimum of 4 h of fasting, blood sugar was ≤11.0 mmol/L and activity of ^18^F-FDG injected was calculated based on weight using the formula: [(body weight ÷ 10) + 1] × 37 MBq. Vertex-to-mid-thigh imaging was commenced after 60 min of uptake time. A separate lower limb imaging was done if the initial primary lesion was from the lower limb. PET acquisition was in 3D mode at 3 min per bed position. Except where a contraindication existed, CT was done with intravenous contrast using non-ionic contrast material (Omnipaque) injected at a rate of 2 mL/second. Images were reconstructed using OSEM (ordered subsets expectation maximization) to yield axial, sagittal and coronal slices of PET, CT and fused PET/CT images. Both attenuation-corrected and non-corrected images were reviewed for interpretation. CT data was used for the attenuation correction of PET data according to camera manufacturer specifications. A diagnostic CT with intravenous and/or oral contrast agents was done according to our departmental protocols and guidelines and was in line with EANM procedure guidelines, which were followed by our department [12].

### 2.3. Image Interpretation and PET/CT Data Analysis

Image interpretation was performed by two experienced nuclear medicine physicians. The reconstructed images were displayed on a dedicated workstation equipped with syngo software (Siemens Medical Solutions, Buffalo Grove, IL, USA).

A positive finding was defined as a focus of increased ^18^F-FDG uptake as compared with surrounding normal tissue corresponding to the primary tumor site and metastatic disease. Increased ^18^F-FDG uptake due to normal physiology or benign uptake was excluded from the analysis. Benign disease was characterized as a disease that did not anatomically represent metastatic disease, e.g., inflammatory lung changes.

Metabolic PET parameters (maximum standardized uptake value (SUVmax), metabolic tumor volume (MTV) and total lesion glycolysis (TLG)) of the primary tumor, as well as whole-body MTV and TLG of the metastatic disease, were measured. To obtain SUVmax, we drew a semi-automatic spherical volume of interest (VOI) around the primary tumor on PET images. We used an SUV threshold of 2.5 and a 3D isocontour of 41%. MTV and SUVmean were automatically computed by the software from the VOI. The TLG was calculated as the MTV multiplied by the SUVmean of the lesion (TLG = MTV × SUVmean). Whole-body MTV was calculated by adding the sum of all the VOI of the metastatic lesions (whole-body MTV = Σ (VOI of all metastatic lesions)), which were obtained on PET images. Whole-body TLG was calculated by adding the sum of all whole-body MTVs and SUVmeans of all the metastatic lesions (whole-body TLG = Σ (all whole-body MTVs and SUVmeans of all metastatic lesions)). ^18^F-FDG PET metabolic parameters were measured as previously described in our facility [13,14,15]. Findings on the images were verified using a combination of histological confirmation and medical records.

### 2.4. Clinical Endpoints and Follow-Ups

Overall survival was the time from PET/CT date to the date of death due to any cause or the last time the patient was known to be alive. Patient files were reviewed from the referral clinics that sent them for PET/CT. Overall survival was measured in months, as the patient follow-up in our institution is every 3 months, depending on the condition of the patient. Should patients need an additional follow-up, they may present earlier, and should their conditions remain stable, follow-up is increased to 6 monthly and yearly intervals.

### 2.5. Statistical Analysis

Time-dependent receiver operating characteristic (ROC) curve analysis was performed to determine the optimal cut-off values for the prediction of death. However, the optimal values selected by this method did not give a clear cut-off as the cut-off of choice gave unbalanced data. As a result, a median point was used to create bivariate data for the PET parameters. Numerical data are expressed as median (interquartile range (IQR)). Since the data was non-normal, the Mann–Whitney test was used to compare the median values of the SUVmax, MTV, TLG, whole-body MTV and whole-body TLG between the survivors and the non-survivors. Categorical data were summarized using frequencies and percentages. Survival curves for OS were constructed using the Kaplan–Meier method and the log-rank test was used to determine as mortality rates significant differences between groups of the PET bivariate variables. Univariate and multivariate Cox proportional hazards models were fitted for OS to determine independent demographic and clinicopathologic prognostic factors for OS. Cox models results are expressed as a hazard ratio (HR) with corresponding 95% confidence intervals (CI). Statistical significance was set at 5%. Statistical analyses were performed using the STATA package (version 16).

## 3. Results

### 3.1. Patient Characteristics

This study focused on patients who had restaging PET/CT scans and met the inclusion criteria. Their demographic and clinicopathologic characteristics are shown in Table 1.

A total of 167 patients presented for restaging; after excluding those that did not meet the inclusion criteria, 49 patients remained. This study consisted of 23 (46.9%) females and 26 (53.1%) males. Most of the patients (61.22%, n = 30) were less than 65 years old. The lower limb primary tumors were the most common, observed in 42.86% (n = 21) of the patients; the remainder were distributed in the upper limbs, head and neck, chest and back. The histological types reported among these patients included unspecified malignant melanoma present in 71.43% (n = 35) patients and nodular type (18.37%, n = 9). The majority of the patients had stage IV disease (61.22%, n = 30).

The AJCC clinical stage at the time of referral was documented. Treatment received by the patients prior to the restaging PET was surgery (n = 36) and chemotherapy (n = 13). Patients were then restaged based on clinical and imaging findings. Patients in stages II and III were upstaged and those in stage IV either progressed or had stable disease. Based on the new stages, nine patients proceeded to receive immunotherapy, three received chemotherapy and the rest were followed up by observation and/or referral to palliative care.

The median follow-up time for survival was 12 months (interquartile range (IQR) of 3–32 months). High mortality rates were observed among males, those aged <65 years and those in clinical stage IV.

### 3.2. Metabolic PET Parameters

The median (IQR) SUVmax, MTV, TLG, whole-body MTV and whole-body TLG for the patients were 6.62 (3.1–12.6), 8.06 cm^3^ (2.9–21.0), 19.49 (6.8–118.3), 33.36 (10.2–100.2) and 462.89 (59.3–1553.3), respectively. Compared to the survivors, non-survivors had a significantly higher median MTV (11.86 cm^3^ vs. 5.68 cm^3^; *p*-value = 0.022), TLG (31.25 vs. 14; *p*-value = 0.0357), whole-body MTV (53.9 cm^3^ vs. 14.4 cm^3^; *p*-value = 0.0076) and whole-body TLG (963.4 vs. 114.6; *p*-value = 0.0056) (Table 2).

### 3.3. Survival Analysis

**Figure 1:**
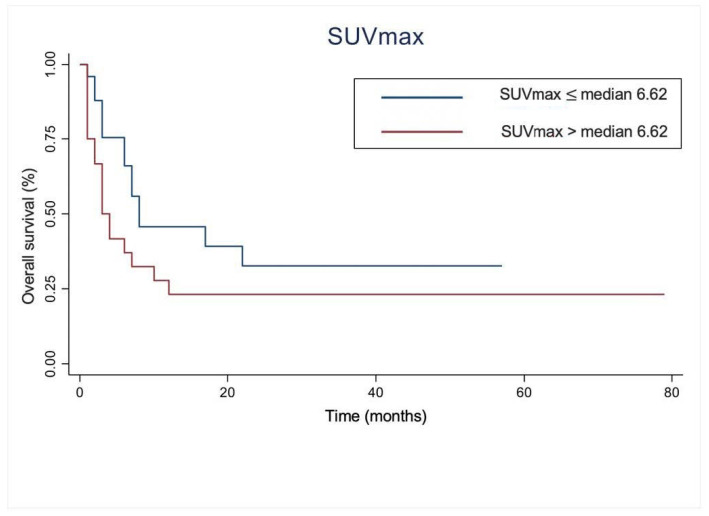
Kaplan–Meier curves for overall survival in restaging melanoma with respect to maximum standard uptake value (SUVmax).

The Kaplan–Meier plots showed that there was no significant difference between patients with SUVmax ≤ median (6.62) compared to those with SUVmax > median (6.62), *p*-value = 0.0905 (Figure 1).

**Figure 2:**
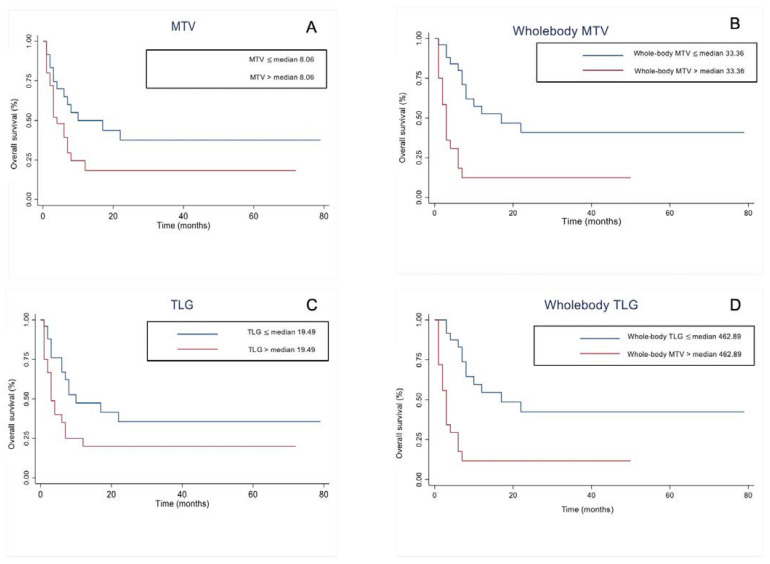
Kaplan–Meier curves for overall survival in restaging melanoma with respect to metabolic tumor volume (MTV) (**A**), whole-body MTV (**B**), total lesion glycolysis (TLG) (**C**), and whole-body TLG (**D**).

**Figure 3:**
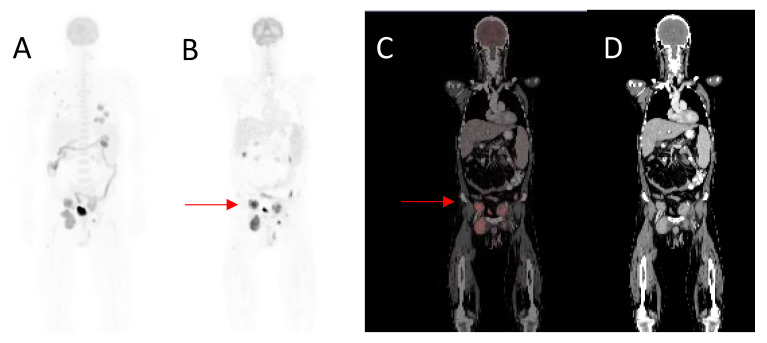
A 50-year-old male, diagnosed with malignant melanoma of the right groin with inguinal lymph node metastases, excision and resection of metastatic inguinal nodes, presented with right groin recurrence. Maximal intensity projection image (**A**), coronal ^18^F-FDG- PET (**B**), fused (**C**) and CT (**D**) images demonstrating right inguinal recurrence (arrow). He also had abdominal and retroperitoneal lesions. MTV 174.59 cm^3^, TLG 1611.46, SUVmax 22.12, whole-body MTV 780.55 cm^3^ and whole-body TLG 28,279.33. Overall survival was 5 months.

**Figure 4:**
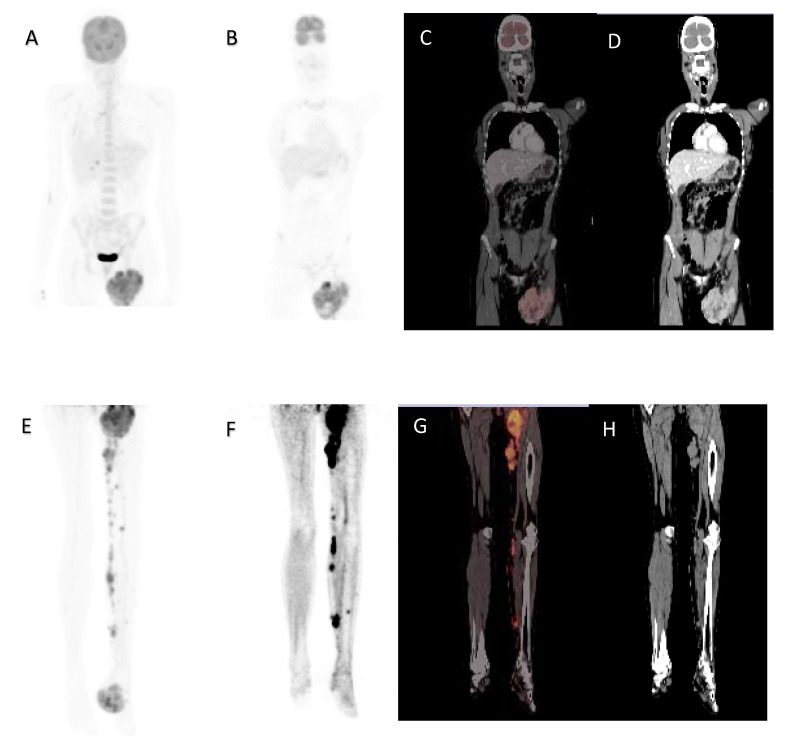
A 32-year-old female, acral lentiginous melanoma resected from the left foot, presented with a recurrence and nodal metastases. Maximal intensity projection image (**A**), coronal ^18^F-FDG PET (**B**), fused (**C**) and CT (**D**) images demonstrating large inguinal node metastases. Maximal intensity projection image (**E**), Coronal ^18^F-FDG PET of lower limbs (**F**), fused (**G**) and CT (**H**) images demonstrating left foot primary with subcutaneous and nodal metastases in the left leg. MTV 126.73 cm^3^, TLG 567.75, SUVmax 10.78, whole-body MTV 635.48 cm^3^ and whole-body TLG 9964.33. Overall survival was 8 months.

The bivariate log-rank test showed significant difference in predicting survival using the log-rank test: MTV ≤ median (8.06 cm^3^) vs. MTV > median (8.06 cm^3^) (*p*-value = 0.0506); TLG ≤ median (19.49) vs. TLG > median (19.49) (*p*-value < 0.0291); whole-body MTV ≤ median (33.36 cm^3^) vs. whole-body MTV > median (33.36 cm^3^) (*p*-value = 0.0001) and whole-body TLG ≤ median (462.89) vs. whole-body TLG > median (462.89) (*p*-value < 0.001). Higher MTV, TLG, whole-body MTV and whole-body TLG were associated with lower survival (see Figure 2, Figure 3 and Figure 4).

### 3.4. Univariate Analysis of Demographic and Clinicopathology in Relation to Overall Survival

In relation to overall survival, patients aged >65 years had a 1.88-fold statistically significant increased risk of death compared to those who were aged <65 years (*p*-value = 0.08). Male patients had a 2.26-fold increased risk of death compared to females (*p*-value = 0.027). Patients with MTV > 12.39 cm^3^ were 2.53 times more likely to die compared to those with MTV ≤ 12.39 cm^3^ (*p*-value = 0.01), while patients with TLG > 36.84 had an increased risk factor of 2.43 compared to TLG ≤ 36.84 (*p*-value < 0.014). The risk of mortality was 4.09 times higher amongst patients with a whole-body MTV > 51.15 cm^3^ compared to those with a whole-body MTV ≤ 51.15 cm^3^ (*p*-value < 0.001), while patients with a whole-body TLG > 564.47 were 4.33 times more likely to die compared to those with a whole-body TLG ≤ 564.47 (*p*-value < 0.001). This is shown in Table 3.

## 4. Discussion

In this study, we evaluated the significance of metabolic ^18^F-FDG PET/CT volumetric parameters in predicting the overall survival of patients presenting for restaging. Our patient cohort had a recurrence in the primary site of disease, as well as distant disease. Regarding the multivariate and univariate parameters, patients that demonstrated higher MTV and TLG were at a higher risk of death. In relation to survival, only whole-body TLG was significantly associated. Our data did not demonstrate a positive correlation with other variables, such as age or the site of the primary tumor, and survival.

Metabolic PET parameters have been discussed as a potential benefit in the prognosis of solid tumors. This has been seen in head and neck cancers, lung cancer and gynecological malignancies [16]. There are several metabolic parameters measured in ^18^F-FDG PET/CT. A high metabolic volume in ^18^F-FDG PET scans is associated with poor prognosis and changes in metabolic activity can be used to monitor response to therapy [17]. Metabolic PET parameters have been demonstrated to be independent prognostic factors for progression-free survival (PFS) and overall survival (OS) [18]. Metabolic PET parameters that are tumor-based, namely, MTV and TLG, have been shown to better represent the entire tumor and are closely associated with prognosis in various malignancies [18]. MTV and TLG have also been demonstrated to have a greater association with PFS and OS when compared to SUVmax. Metabolic parameters of tumor volumes have been demonstrated to be associated with prognostic values of overall survival and progression-free survival in esophageal cancer [19], head and neck cancer [20] and small cell lung cancer [21].

A study by Son et al. looked at the prognostic relevance of MTV and TLG in patients with malignant melanoma and found that pre-treatment MTV and TLG may be useful in risk-stratifying patients for likelihood of death and recurrence, with TLG being the best predictor [9]. Our findings were similar, where those patients with higher MTV, TLG and whole-body TLG were associated with overall survival. The difference between our study and theirs is that they looked at patients who presented for initial staging and our cohort of patients presented for restaging. Our study did not focus on progression-free survival, as a large proportion of our patients already had stage IV disease. The findings did however agree that metabolic parameters played a role in prognosis.

Another study by Ito et al., which looked at tumor volumes, demonstrated that whole-body MTV is a strong independent prognostic factor in determining which melanoma patients will respond to immunotherapy [8]. Our study did not look at the response to therapy but demonstrated that a higher whole-body MTV was associated with poorer survival. This agrees with another study that looked at treatment response and reported that those with more tumor involvement faired more poorly than those with less tumor involvement. This demonstrates that tumor volumes can be used to see which patients are more likely to benefit from intervention.

More recently, a study by Reinert et al. demonstrated the prognostic value of metabolic parameters in patients with melanoma regarding progression-free and overall survival [22]. This study demonstrated a positive correlation between MTV and TLG with overall and progression-free survival [22]. These findings were almost similar to our findings; however, this was done on a European population that presumably has better access to healthcare. In our population, patients typically present late and with more advanced disease [2]; therefore, in our population, having an additional risk stratification tool may guide clinicians regarding which therapies to use, more frequent follow-ups or earlier palliative treatment if necessary. The study also looked at other parameters, such as serological markers, lactate dehydrogenase and C-reactive protein. These specific parameters were not commonly reviewed in our patient cohort. Prognostication using ^18^F-FDG PET/CT was also reviewed by Schweigoffer-Zwink et al., who demonstrated that metabolic parameters in patients with advanced cutaneous melanoma were predictive for survival in melanoma patients undergoing immunotherapy [23]. This study also found that tumor-to-background values had a stronger predictive value than MTV and TLG. Our study did not look at tumor-to-background ratios but similarly demonstrated that metabolic PET parameters can be predictive in a resource-constrained setting such as ours.

As our study specifically looked at restaging, our findings were somewhat similar to the Albano et al. group in Italy, which reviewed patients with ^18^F-FDG PET/CT after surgery with suspicion for recurrence or metastatic disease post-surgical intervention; they found that imaging a positive scan was associated with an increased risk of disease progression and a negative study demonstrated longer survival than a positive one [24]. Metabolic parameters were not reviewed in this study; however, findings agree that a positive PET has prognostic outcomes in survival [24]. ^18^F-FDG PET/CT also has the added advantage that it can detect melanoma recurrence in asymptomatic patients prior to clinical detection and this was demonstrated in a study done at our center [25].

Survival in melanoma is dependent on the stage at diagnosis. The different factors that encompass staging, namely, tumor size, nodal involvement and metastases, have been evaluated for melanoma-specific survival. Overall survival tends to be poorer depending on the stage at diagnosis [26]. This correlates with our data, as most of our patients presented for restaging at a later stage and had poorer outcomes. Due to mostly delayed presentation in our patient population, our study evaluated patients that demonstrated disease in the primary tumor at restaging with distant metastases. Our patients had significantly higher tumor volumes compared to what has been described by other authors [11,22].

The strength of this study was in demonstrating the value of metabolic parameters ^18^F-FDG PET/CT in restaging patients with malignant melanoma for prognostic purposes. The role in recurrent melanoma is yet to be defined in a prospective study and would be beneficial to guide clinicians on potential clinical outcomes of patients, especially in recurrent disease.

The limitations of this study are that it is retrospective with a limited sample size of patients that presented to our hospital for restaging. Our study sample was based on the hospital records, which were not intended for research; therefore, challenges with incomplete records were encountered. A large proportion of our patients were also lost to follow-up, which also influenced our results. Difficulties in record keeping have been described in a resource-constrained setting similar to ours by Pirkle et al., who found that researchers in diverse settings struggle with record keeping. The authors mentioned that illegible notes or missing records can affect hospital care and research and mentioned the need for electronic records, which may assist with improving this [27]. Unfortunately, in our setting, medical records are still done on paper and only imaging is available electronically. A study done in a first-world setting, namely, in Taiwan, by Li et al. found that, although electronic medical records were available, most retrospective studies had a case number of fewer than 100 patients, with the average being 41 [28]. The lower case numbers were speculated to be due to the authors’ preference of accessing paper records despite the availability of electronic records [28]. These studies demonstrate that lower case numbers in retrospective data are not unique to our population alone, but are seen in low-income and first-world countries with access to better record keeping [27,28]. Another limitation is the inability to correlate the histology of all the metastatic lesions to truly confirm melanoma metastases despite anatomical features of metastases. Our data demonstrated a positive correlation with tumor volumes and overall survival in this retrospective analysis of patients presenting for restaging. Prospective studies in patients with melanoma preventing for staging would be beneficial, as the bulk of known literature is retrospective.

## 5. Conclusions

In this patient cohort that presented for restaging, we found that a high MTV and TLG of the primary tumor and whole-body TLG were prognostic for overall survival. These findings may assist clinicians in evaluating and recognizing patients with a poorer prognosis in a similar population group.

## Figures and Tables

**Table 1:** Patient demographic and clinicopathologic characteristics.

Variables	Restaging		
	Survivors n (%)	Non-Survivors n (%)	Total n (%)
Age (years)			
<65	13 (76.47)	17 (53.13)	30 (61.22)
≥65	4 (23.53)	15 (46.88)	19 (38.78)
Sex			
Female	11 (64.71)	12 (37.5)	23 (46.94)
Male	6 (35.29)	20 (62.5)	26 (53.06)
Site of the primary tumor			
Upper limb	2 (11.76)	2 (6.25)	4 (8.16)
Lower limb	7 (41.18)	14 (43.75)	21 (42.86)
Head and neck	4 (23.53)	12 (37.5)	16 (32.65)
Chest and back	4 (23.53)	4 (12.5)	8 (16.33)
Histology			
Acral lentiginous	0	1 (3.13)	1 (2.04)
Amelanotic	2 (11.76)	0	2 (4.08)
Choroidal melanoma	0	1 (3.13)	1 (2.04)
Malignant melanoma	9 (52.94)	26 (81.25)	35 (71.43)
Nodular	5 (29.41)	4 (12.5)	9 (18.37)
Superficial spreading	1 (5.88)	0	1 (2.04)
Clinical staging			
II	3 (17.65)	2 (6.25)	5 (10.2)
III	8 (47.06)	6 (18.75)	14 (28.57)
IV	6 (35.29)	24 (75.0)	30 (61.22)

**Table 2:** Metabolic PET parameters of survivors and non-survivors.

PET Parameter	Restaging Group			
	Total Median (IQR)	Survivors Median (IQR)	Non-Survivors Median (IQR)	*p*-Value
SUVmax	6.62 (3.1–12.6)	3.66 (2.9–8.7)	8.65 (3.27–13.6)	0.0661
MTV (cm^3^)	8.06 (2.9–21.0)	5.68 (2.5–12.2)	11.86 (4.1–191.6)	0.022
TLG	19.49 (6.8–118.3)	14.3 (5.0–23.9)	31.25 (9.9–191.6)	0.0357
Whole-body MTV (cm^3^)	33.36 (10.2–100.2)	14.4 (8.3–34.7)	53.91 (21.4–547.7)	0.0076
Whole-body TLG	462.89 (59.3–1553.3)	114.6 (23.9–479.5)	963.44 (167.9–11523.2)	0.0056

IQR: Interquartile range; SUVmax: maxiumum standard uptake value; MTV: metabolic tumor volume; TLG: total lesion glycolysis.

**Table 3:** Univariate analysis of demographic and clinicopathology in relation to overall survival.

Variables	Overall Survival	
	HR (95%CI)	*p*-Value
Age (years)		
<65	1 (base)	
≥65	1.88 (0.93.82)	0.08
Sex		
Female	1 (base)	
Male	2.26 (1.09–4.67)	0.027
Clinical staging		
2	1 (base)	
3	1.21 (0.24–5.99)	0.818
4	3.41 (0.800–14.57)	0.097
SUVmax		
≤8.61	1 (base)	
>8.61	1.78 (0.88–3.58)	0.108
MTV (cm^3^)		
≤12.39	1 (base)	
>12.39	2.53 (1.25–5.15)	0.01
TLG		
≤36.84	1 (base)	
>36.84	2.43 (1.19–4.91)	0.014
Whole-body MTV (cm^3^)		
≤51.15	1 (base)	
>51.15	4.09 (1.97–8.48)	<0.001
Whole-body TLG		
≤564.47	1 (base)	
>564.47	4.33 (2.05–913)	<0.001

HR: Hazards ratio; CI: Confidence interval.

## Data Availability

The data presented in this study are available on request from the corresponding author. The data is not publicly available due to patient confidentiality.
